# Supplementary material for: ZFP281-BRCA2 prevents R-loop accumulation during DNA replication
Source: Nat Commun. 2022 Jun 17;13:3493. doi: 10.1038/s41467-022-31211-9 (PMC9205938; doi:10.1038/s41467-022-31211-9)
Supplement: Supplementary file 1 — Supplementary Information [file 41467_2022_31211_MOESM1_ESM.pdf]

ZFP281-BRCA2 prevents R-loop accumulation during DNA replication

SUPPLEMENTARY INFORMATION

Supplementary Figures

Supplementary Figure 1-14

Supplementary Table

Supplementary Figures

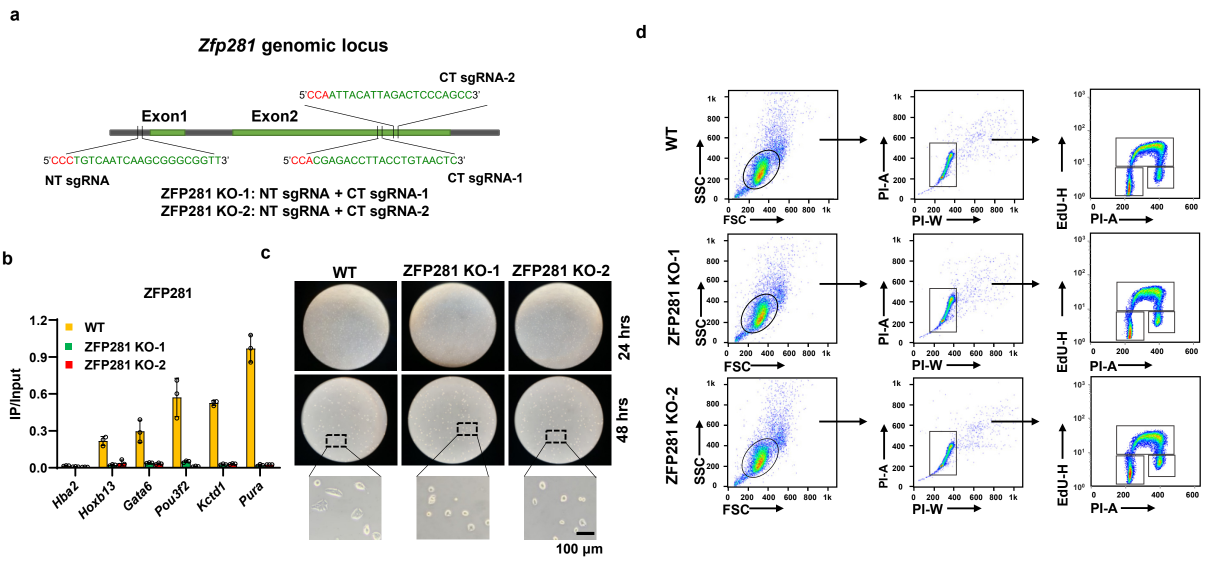

Supplementary Figure 1. ZFP281 KO in mouse ES cells. a, Schematic representation of the positions of the three sgRNAs used to delete ZFP281. **b,** ChIP-qPCR showing that the binding of ZFP281 to the tested regions is completely abolished in the two established ZFP281 KO ES cells. The *HEMO* gene (*Hba2*) serves as a negative control for ChIP-qPCR. Mean ± SEM from three independent experiments. **c,** Morphology of WT, ZFP281 KO-1 and ZFP281 KO-2 ES cell clones after culturing for 24 and 48 hrs. **d**, FACS gating strategy used for Figure 1c. Source data are provided as a Source Data file.

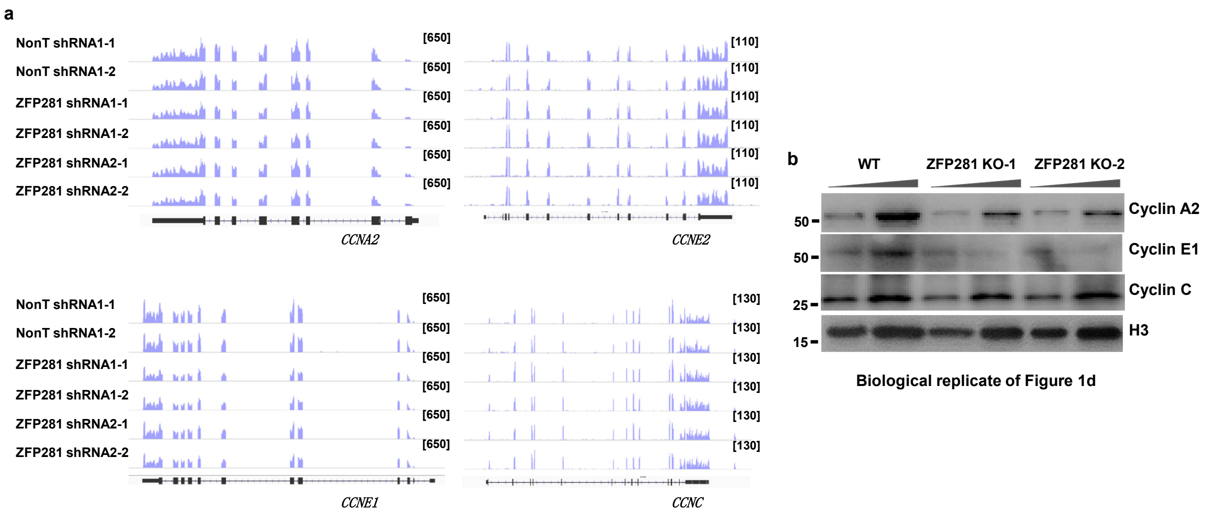

Supplementary Figure 2. RNA and protein levels of Cyclin A2, Cyclin E and Cyclin C after ZFP281 depletion. a, Genome browser track files showing RNA levels of Cyclin A2 (*CCNA2*), Cyclin E (*CCNE1* and *CCNE2*) and Cyclin C (*CCNC*) in control and ZFP281 knockdown cells. **b,** Western blot analysis showing the levels of Cyclin A2, Cyclin E1 and Cyclin C in WT, ZFP281 KO-1, and ZFP281 KO-2 ES cells. Histone H3 was used as a loading control. Shown is one of the biological replicates for Figure 1d. Source data are provided as a Source Data file.

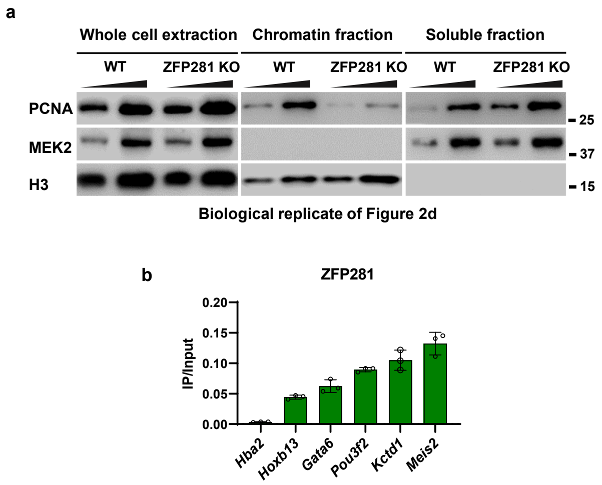

Supplementary Figure 3. ZFP281 binds to nascent DNA and recruits PCNA to chromatin. a, Western blot analysis showing the levels of total, chromatin-bound and soluble γH2A.X and PCNA in WT and ZFP281 KO ES cells. Histone H3 and MEK2 were used as loading controls. Shown is one of the biological replicates for Figure 2d. **b,** ChIP-qPCR showing the binding of ZFP281 to the tested regions used in the re-ChIP assay in Figure 2f. The *HEMO* gene (Hba2) serves as a negative control for ChIP-qPCR. Mean ± SEM from three independent experiments. Source data are provided as a Source Data file.

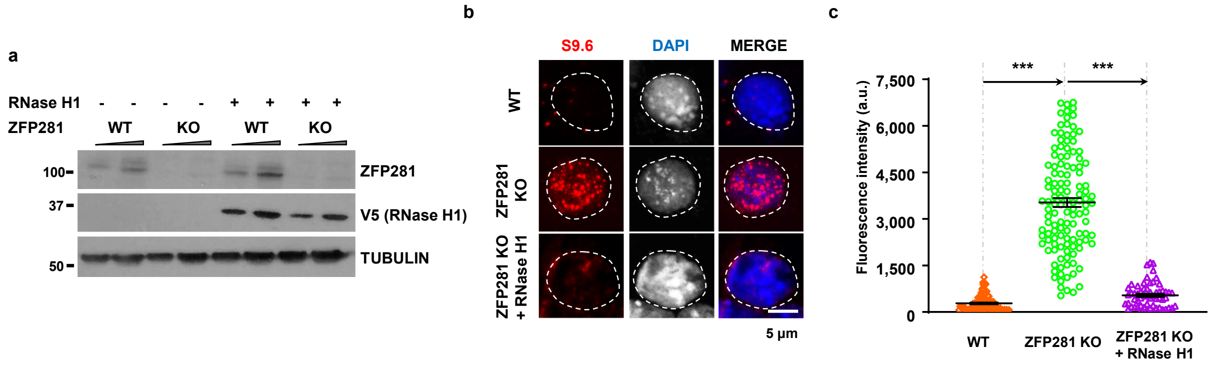

Supplementary Figure 4. Removal of aberrant R-loop accumulation in ZFP81 KO cells by overexpressing RNase H1. a, Western blot analysis showing the overexpression of V5 tagged RNase H1 in WT and ZFP281 KO ES cells. α-TUBULIN was used as a loading control. **b,** Representative immunofluorescence images with the S9.6 antibody showing the levels of R-loop in WT, ZFP281 KO, and ZFP281 KO with RNase H1 overexpression ES cells. DNA was counterstained using DAPI. **c,** Fluorescence intensities of R-loop loci in WT (total n = 95), ZFP281 KO (total n = 123), and ZFP281 KO with RNase H1 overexpression (total n = 59) ES cells. Error bars represent 95% confidence intervals. Two-tailed, unpaired Student's *t* tests were performed. (WT vs. ZFP281 KO, $p < 0.0001$; ZFP281 KO vs. ZFP281 KO + RNase H1 , $p < 0.0001$.) $*p < 0.05$, $**p < 0.01$, $***p < 0.001$, n.s. = not significant. The experiments in (**a**) were performed five times, and (**b**) three times with similar results. Source data are provided as a Source Data file.

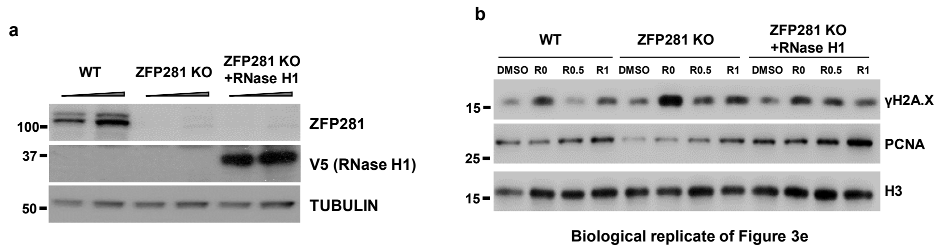

Supplementary Figure 5. Removal of aberrant R-loop accumulation in ZFP81 KO cells rescues DNA replication defects caused by ZFP281 knockout. a, Western blot analysis showing the overexpression of V5 tagged RNase H1 in WT and ZFP281 KO ES cells. α-TUBULIN was used as a loading control. **b,** Western blot analysis showing the levels of chromatin-bound γH2A.X and PCNA in WT, ZFP281 KO, and ZFP281 KO with RNase H1 overexpression ES cells after release from APH treatment for 0, 0.5 and 1 hr respectively. Histone H3 was used as a loading control. Shown is one of the biological replicates for Figure 3e. The experiments in (**a**) were repeated at least five times, and (**b**) three times with similar results. Source data are provided as a Source Data file.

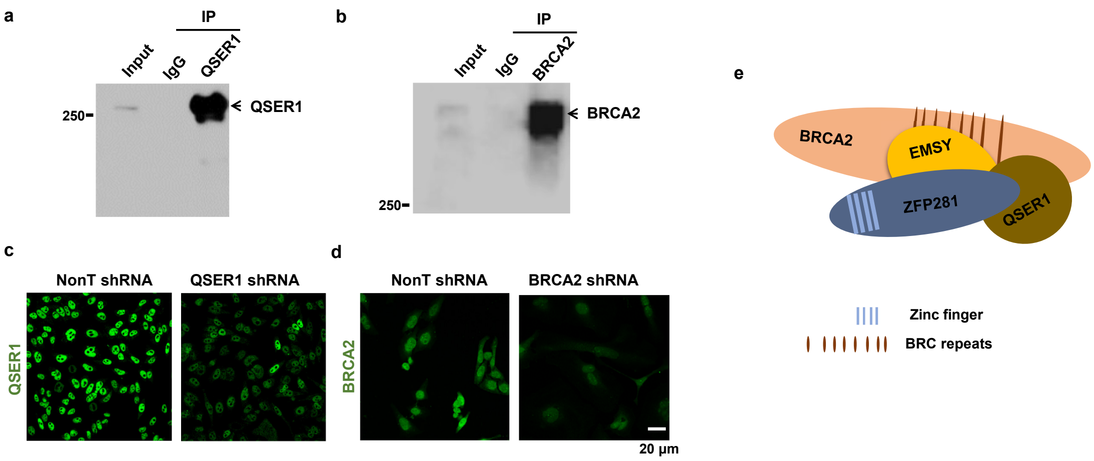

Supplementary Figure 6. Validation of antibodies against QSER1 and BRCA2. a, b, Endogenous immunoprecipitations showing that antibodies against QSER1 (**a**) and BRCA2 (**b**) are specific. **c, d,** Immunofluorescence assays in QSER1 KD (**c**) or BRCA2 KD (**d**) cells showing that antibodies against QSER1 and BRCA2 are specific. **e,** Cartoon model illustrating the interactions among ZFP281, BRCA2, QSER1 and EMSY. The experiments in (**a**-**d**) were performed at least ten times with similar results. Source data are provided as a Source Data file.

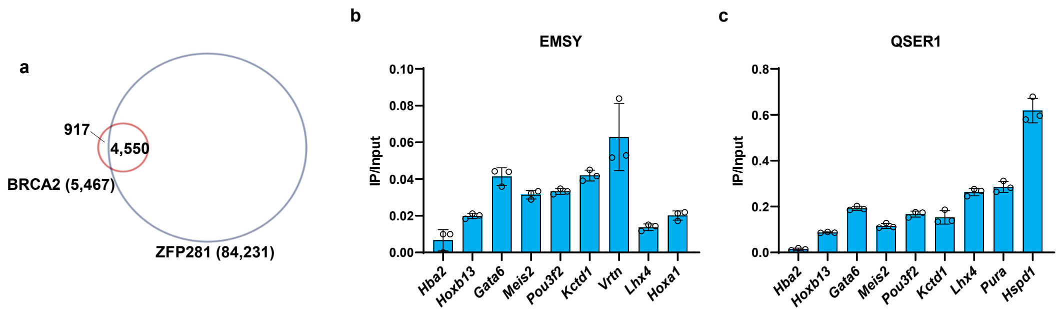

Supplementary Figure 7. Co-occupation of a subsets of genomic regions in mouse ES cells by ZFP281, BRCA2, EMSY and QSER1. a, Venn diagram showing the overlap between ZFP281 and BRCA2 binding sites in mouse ES cells. **b, c,** ChIP-qPCR showing that EMSY (**b**) and QSER1 (**c**) also binds to the tested ZFP281 and BRCA2 co-bound regions. The *HEMO* gene (Hba2) serves as a negative control for ChIP-qPCR. Mean ± SEM from three independent experiments. Three independent experiments show similar results in **b** and **c**. Source data are provided as a Source Data file.

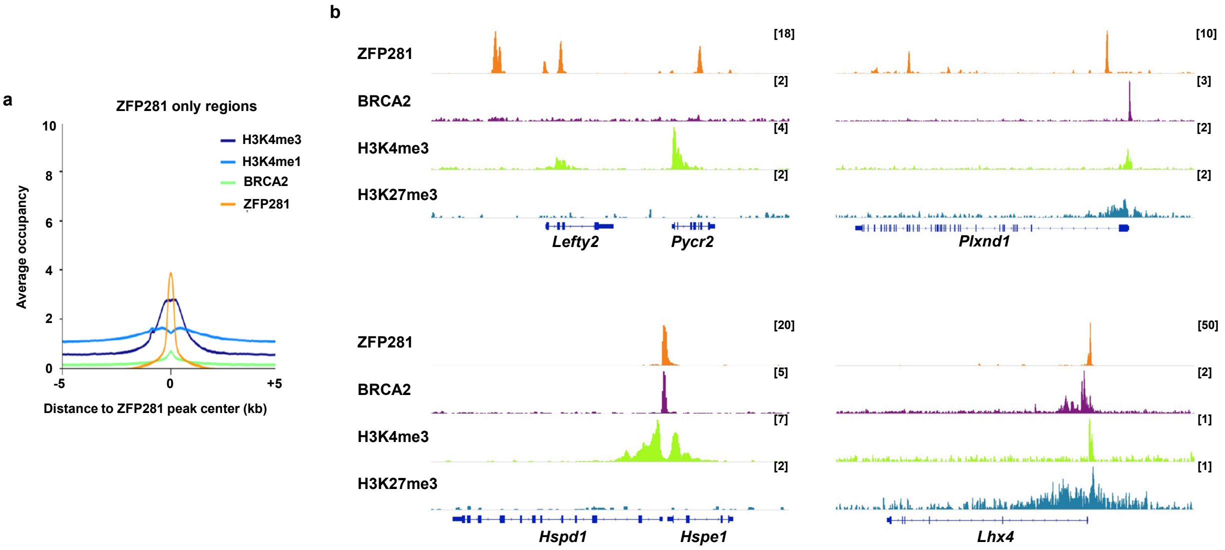

Supplementary Figure 8. Co-occupation of G/C rich promoters by BRCA2 and ZFP281 in mouse ES cells. a, Average occupancy plots of ZFP281, BRCA2, H3K4me1 and H3K4me3 at the ZFP281 only regions in mouse ES cells. Shown are +/− 5 kb of the center of ZFP281 peaks. **b,** ChIP-Seq genome browser track showing the localization of ZFP281, BRCA2, H3K4me3 and H3K27me3 at the ZFP281 only region (upper left panel), the BRCA2 only region (upper right panel), the ZFP281 and BRCA2 co-bound active region (lower left panel), and the ZFP281 and BRCA2 co-bound bivalent region (lower right panel).

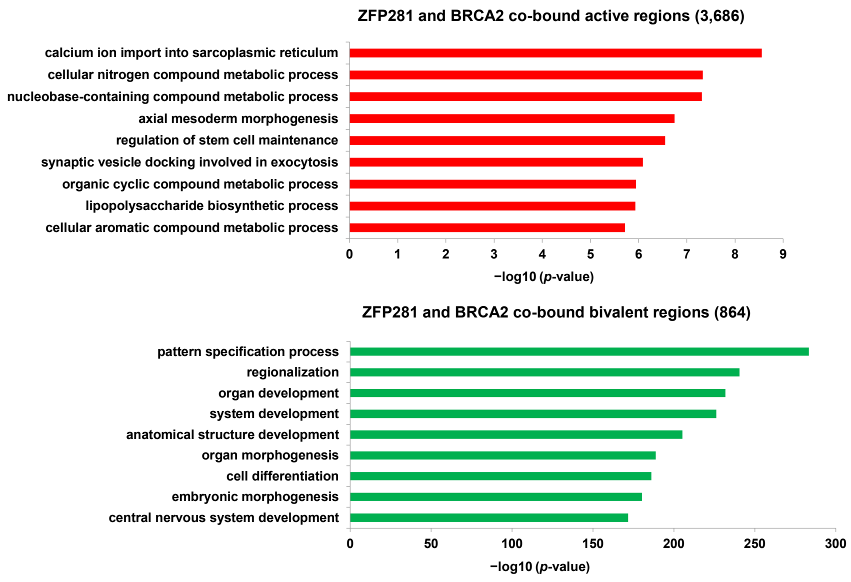

Supplementary Figure 9. Functional annotation of the ZFP281 and BRCA2 co-bound peaks. Functional annotation of the ZFP281 and BRCA2 co-bound peaks in the active group (upper panel) and the bivalent group (lower panel), as reported by GREAT[69]. P-value was used to determine significance of enrichment of terms. *p* value from one-sided Fisher Exact tests.

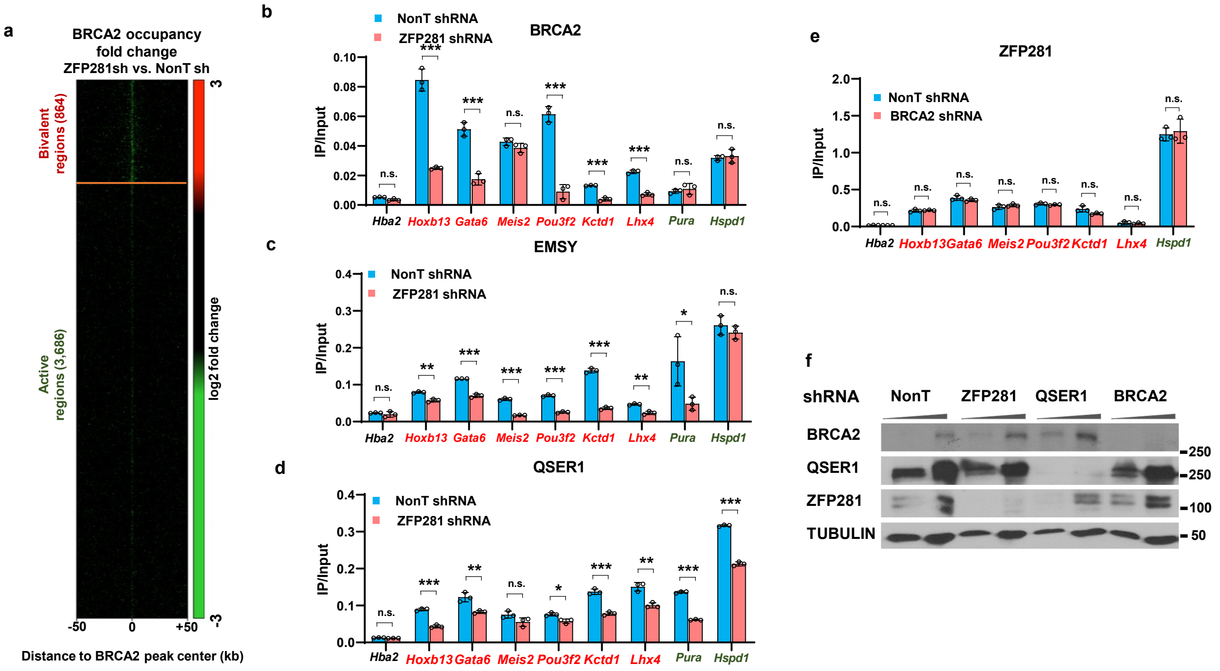

Supplementary Figure 10. Requirement of ZFP281 for the recruitment of BRCA2 to the bivalent regions. a, BRCA2 occupancy log2 fold change after ZFP281 knockdown was measured at the BRCA2 and ZFP281 co-bound regions. Shown are +/− 50 kb around the center of BRCA2 peaks. **b, c, d,** ChIP-qPCR showing that the occupancies of BRCA2 (**b**), EMSY (**c**) and QSER1 (**d**) are reduced at the tested bivalent regions (labelled with red color) after ZFP281 knockdown in mouse ES cells, while remains unchanged at the tested active regions (labelled with green color). **e,** ChIP-qPCR showing that the occupancies of ZFP281 remains unchanged at the regions after BRCA2 knockdown in mouse ES cells. **b, c, d, e,** The *HEMO* gene (Hba2) serves as a negative control for ChIP-qPCR. Mean ± SEM from three independent experiments. Multiple *t* tests were performed. (**b**, *Hba2*, $p = 0.0673$; *Hoxb13*, $p = 0.0002$; *Gata6*, $p = 0.0006$; *Meis2*, $p = 0.1483$; *Pou3f2*, $p = 0.0002$; *Kctd1*, $p = 0.0002$; *Lhx4*, $p = 0.0001$; *Pura*, $p = 0.5120$; *Hspd1*, $p = 0.6794$. **c**, *Hba2*, $p = 0.4570$; *Hoxb13*, $p = 0.0014$; *Gata6*, $p = 0.0001$; *Meis2*, $p < 0.0001$; *Pou3f2*, $p < 0.0001$; *Kctd1*, $p < 0.0001$; *Lhx4*, $p = 0.0013$; *Pura*, $p = 0.0452$; *Hspd1*, $p = 0.3326$. **d**, *Hba2*, $p = 0.0577$; *Hoxb13*, $p = 0.0001$; *Gata6*, $p = 0.0068$; *Meis2*, $p = 0.0796$; *Pou3f2*, $p = 0.0119$; *Kctd1*, $p = 0.0003$; *Lhx4*, $p = 0.0030$; *Pura*, $p < 0.0001$; *Hspd1*, $p < 0.0001$. **e**, *Hba2*, $p = 0.5959$; *Hoxb13*, $p = 0.7372$; *Gata6*, $p = 0.2389$; *Meis2*, $p = 0.3315$; *Pou3f2*, $p = 0.2858$; *Kctd1*, $p = 0.0545$; *Lhx4*, $p = 0.5671$; *Hspd1*, $p = 0.7077$.) $*p < 0.05$, $**p < 0.01$, $***p < 0.001$, n.s. = not significant. **f**, Western blot analysis showing the levels of indicated factors in control and the knockdown cells. α-TUBULIN was used as a loading control. The experiments in (**b**-**f**) were performed three times with similar results. Source data are provided as a Source Data file.

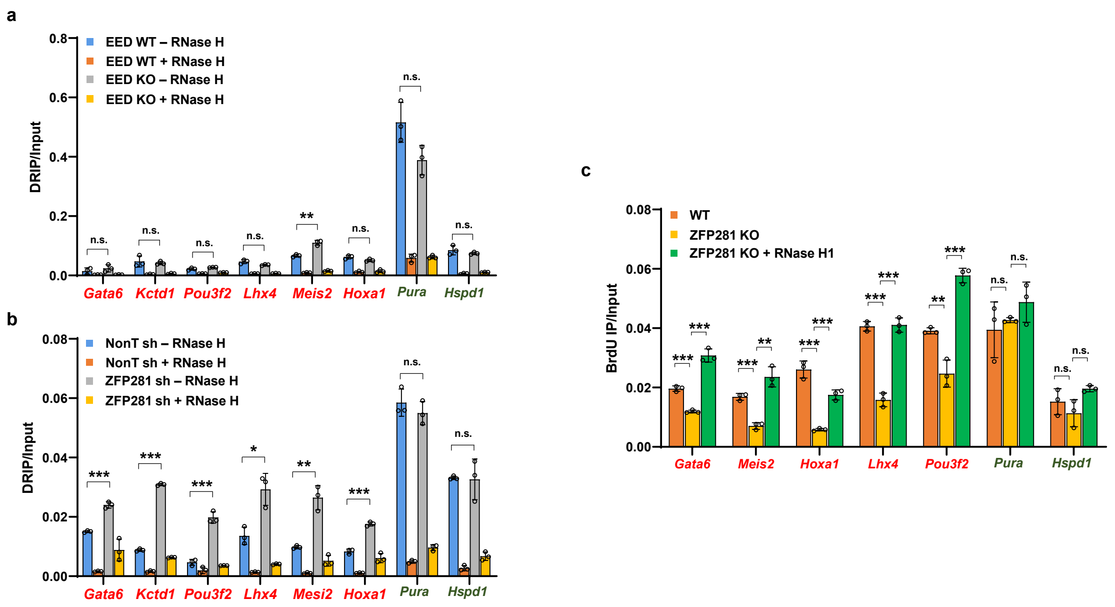

Supplementary Figure 11. ZFP281 knockdown leads to R-loop accumulation at the bivalent regions. **a,** DRIP-qPCR showing that the levels of R-loop are unchanged at the tested regions after EED knockout. **b,** DRIP-qPCR showing that the levels of R-loop are reduced at the tested bivalent regions (labelled with red color) after ZFP281 knockdown, while remains unchanged at the tested active *Pura* and *Hspd1* loci (labelled with green color). DRIP-qPCR analyses in samples pre-treated with RNase H were used as negative controls. sh means shRNA. **c,** BrdU IP-qPCR analyses showing that BrdU incorporation defects at the tested bivalent regions (labelled with red color) in ZFP281 depleted cells after release from APH treatment for 3 hrs can be rescued by RNase H1 overexpression, and that BrdU incorporation at the tested active *Pura* and *Hspd1* loci (labelled with green color) is unaffected by ZFP281 knockout. **a**, **b**, **c**, Mean ± SEM from three independent experiments. Multiple t tests were performed. (**a**, *Gata6*, p = 0.3786; *Kctd1*, p =0.6618; *Pou3f2*, p =0.0858; *Lhx4*, p = 0.0579; *Meis2*, p = 0.0013; *Hoxa1*, p = 0.0611; *Pura*, p =0.0579; *Hspd1*, p = 0.3205. **b**, *Gata6*, p = 0.0002; *Kctd1*, p < 0.0001; *Pou3f2*, p = 0.0003; *Lhx4*, p = 0.0115; *Meis2*, p = 0.0022; *Hoxa1*, p =0.0001; *Pura*, p =0.3655; *Hspd1*, p = 0.9019. **c**, WT vs. ZFP281 KO, *Gata6*, p = 0.0002; *Meis2*, p = 0.0005; *Hoxa1*, p = 0.0003; *Lhx4*, p = 0.00019; *Pou3f2*, p = 0.0059; *Pura*, p = 0.5794; *Hspd1*, p = 0.4316. ZFP281 KO vs. ZFP281 KO + RNase H1, *Gata6*, p = 0.0001; *Meis2*, p = 0.0013; *Hoxa1*, p = 0.0003; *Lhx4*, p = 0.0002; *Pou3f2*, p = 0.0004; *Pura*, p = 0.1996; *Hspd1*, p = 0.0552.) $*p < 0.05$, $**p < 0.01$, $***p < 0.001$, n.s. = not significant. All the above experiments were performed three biological replicates. Source data are provided as a Source Data file.

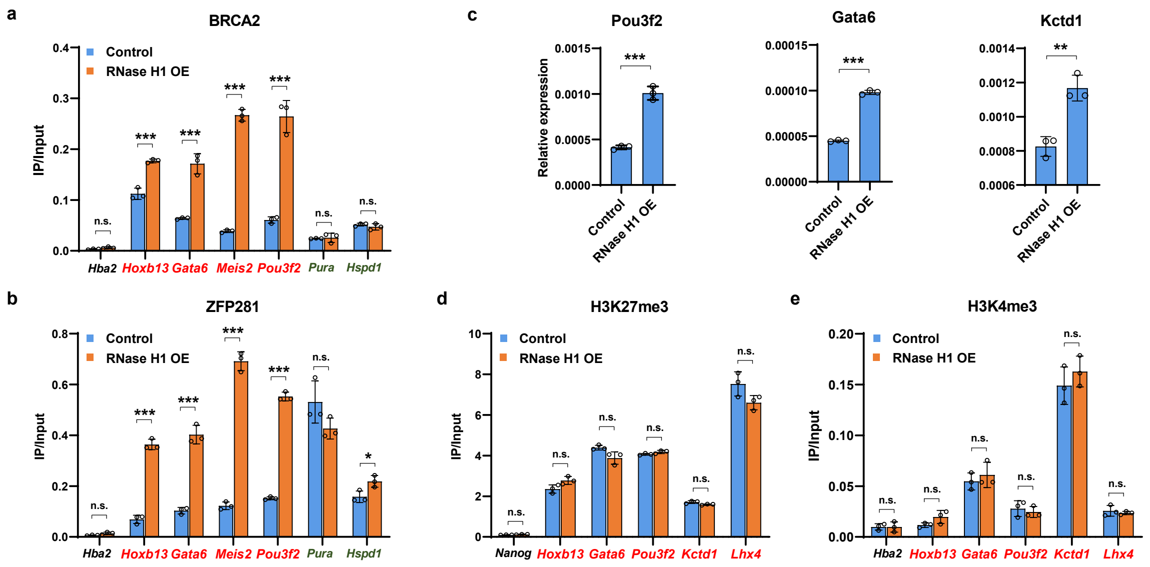

Supplementary Figure 12. Removal of R-loop by overexpressing RNase H1 increases the occupancies of ZFP281 and BRCA2 at the bivalent regions. a, b, ChIP-qPCR showing that the occupancies of BRCA2 (**a**) and ZFP281 (**b**) are increased at the tested bivalent regions (labelled with red color) after RNase H1 overexpression in mouse ES cells, while remains almost unchanged at the tested active regions (labelled with green color). The *HEMO* gene (Hba2) serves as a negative control for ChIP-qPCR. Mean ± SEM from three independent experiments. Multiple *t* tests were performed. (**a**, *Hba2*, $p = 0.0731$; *Hoxb13*, $p = 0.0007$; *Gata6*, $p = 0.0007$; *Meis2*, $p < 0.0001$; *Pou3f2*, $p = 0.0004$; *Pura*, $p = 0.8094$; *Hspd1*, $p = 0.3458$. **b**, *Hba2*, $p = 0.0616$; *Hoxb13*, $p < 0.0001$; *Gata6*, $p = 0.0002$; *Meis2*, $p < 0.0001$; *Pou3f2*, $p < 0.0001$; *Pura*, $p = 0.1234$; *Hspd1*, $p = 0.0319$.) $*p < 0.05$, $**p < 0.01$, $***p < 0.001$, n.s. = not significant. **c,** RT-qPCR showing that the RNA levels of Pou3f2, Gata6 and Kctd1 are slightly increased after RNase H1 overexpression in mouse ES cells. Mean ± SEM from three independent experiments. Two-tailed, unpaired Student's *t* tests were performed. (*Pou3f2*, $p = 0.0002$; *Gata6*, $p < 0.0001$; *Kctd1*, $p = 0.0034$) $*p < 0.05$, $**p < 0.01$, $***p < 0.001$, n.s. = not significant. **d, e,** ChIP-qPCR showing that the occupancies of H3K27me3 (**d**) and H3K4me3 (**e**) remains unchanged at the tested regions after RNase H1 overexpression in mouse ES cells. The *HEMO* gene (*Hba2*) serves as a negative control for ChIP-qPCR. Mean ± SEM from three independent experiments. Multiple *t* tests were performed. (**d**, *Nanog*, $p = 0.0566$; *Hoxb13*, $p = 0.0597$; *Gata6*, $p = 0.0506$; *Pou3f2*, $p = 0.0937$; *Kctd1*, $p = 0.0508$; *Lhx4*, $p = 0.0822$.**e**, *Hba2*,

p = 0.9975; *Hoxb13*, *p* = 0.1119; *Gata6*, *p* = 0.5023; *Pou3f2*, *p* = 0.5728; *Kctd1*, *p* = 0.3700; *Lhx4*, *p* = 0.5452)

$*p < 0.05$, $**p < 0.01$, $***p < 0.001$, n.s. = not significant. Source data are provided as a Source Data file.

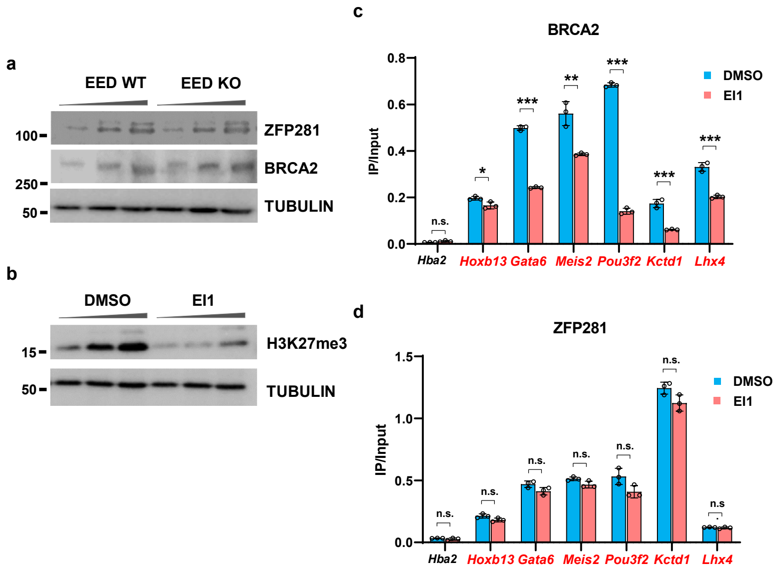

Supplementary Figure 13. Requirement of PRC2 for the recruitment of BRCA2 to the bivalent regions. **a,** Western blot analysis of BRCA2 and ZFP281 in EED WT and EED KO ES cells. **b,** Western blot analysis of H3K27me3 in control and EI1-treated ES cells. **a, b,** α-TUBULIN was used as a loading control. **c,** ChIP-qPCR showing that the occupancies of BRCA2 are reduced at the tested bivalent regions in mouse ES cells treated with EI1. **d,** ChIP-qPCR showing that the occupancies of ZFP281 are not obviously changed at the tested bivalent regions in mouse ES cells after EI1 treatment. **c, d,** The *HEMO* gene (*Hba2*) serves as a negative control for ChIP-qPCR. Mean ± SEM from three independent experiments. Multiple t tests were performed. (**c**, *Hba2*, $p = 0.0785$; *Hoxb13*, $p = 0.0324$; *Gata6*, $p < 0.0001$; *Meis2*, $p = 0.0043$; *Pou3f2*, $p < 0.0001$; *Kctd1*, $p = 0.0004$; *Lhx4*, $p = 0.0004$. **d**, *Hba2*, $p = 0.2404$; *Hoxb13*, $p = 0.0732$; *Gata6*, $p = 0.0676$; *Meis2*, $p = 0.0570$; *Pou3f2*, $p = 0.0559$; *Kctd1*, $p = 0.0592$; *Lhx4*, $p = 0.3428$.) $*p < 0.05$, $**p < 0.01$, $***p < 0.001$. The experiments in (**a-b**) were performed three times with similar results. Source data are provided as a Source Data file.

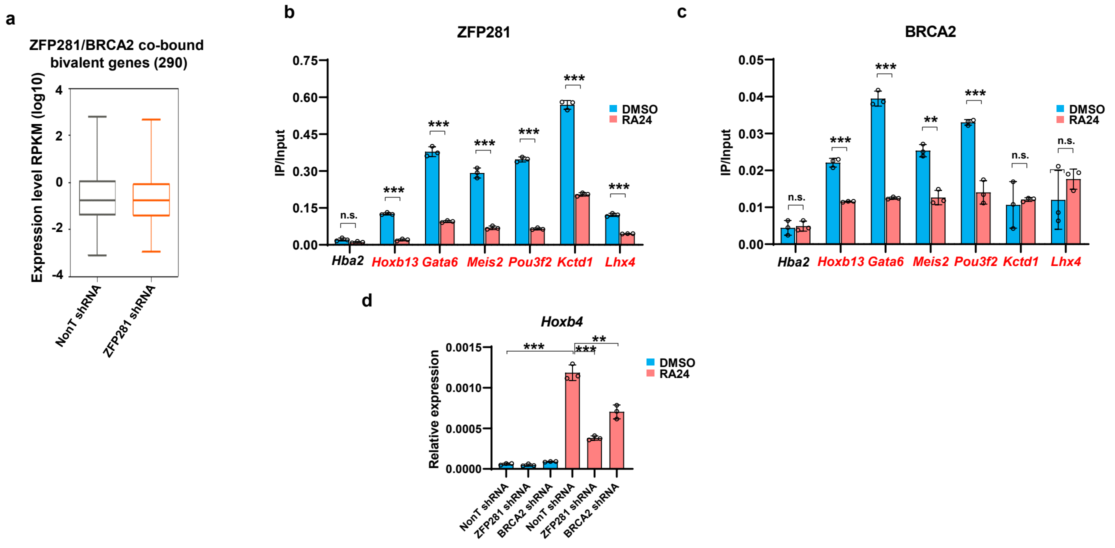

Supplementary Figure 14. Requirement of ZFP281 and BRCA2 for the proper expression of PRC2-bound genes. a, Box plot showing that the expression of the ZFP281 and BRCA2 co-bound genes remains unchanged in ZFP281 KO cells. The horizontal line across the box indicates the median, and the whiskers indicate the minimum and maximum values, box boundaries are the 25th and 75th percentiles. **b, c,** ChIP-qPCR showing that the occupancies of ZFP281 (**b**) and BRCA2 (**c**) are reduced at the tested bivalent regions in mouse ES cells after RA treatment. The *HEMO* gene (*Hba2*) serves as a negative control for ChIP-qPCR. Mean ± SEM from three independent experiments. Multiple *t* tests were performed. (**b**, *Hba2*, $p = 0.0535$; *Hoxb13*, $p < 0.0001$; *Gata6*, $p < 0.0001$; *Meis2*, $p = 0.0001$; *Pou3f2*, $p < 0.0001$; *Kctd1*, $p < 0.0001$; *Lhx4*, $p < 0.0001$. **c**, *Hba2*, $p = 0.7645$; *Hoxb13*, $p = 0.0001$; *Gata6*, $p < 0.0001$; *Meis2*, $p = 0.0010$; *Pou3f2*, $p = 0.0006$; *Kctd1*, $p = 0.7127$; *Lhx4*, $p = 0.3111$.) $*p < 0.05$, $**p < 0.01$, $***p < 0.001$, n.s. = not significant. **d,** RT-qPCR showing that the induction of *Hoxb4* by RA is impaired in ZFP281 KO ES cells. Mean ± SEM from three independent experiments. Two-tailed, unpaired Student's *t* tests were performed. (*Hoxb4* in NonT shRNA , DMSO vs. RA24, p < 0.0001; *Hoxb5* in RA24, NonT shRNA vs. ZFP281 shRNA, $p = 0.0002$; NonT shRNA vs. BRCA2 shRNA, $p = 0.0029$.) $*p < 0.05$, $**p < 0.01$, $***p < 0.001$, n.s. = not significant. Source data are provided as a Source Data file.

Supplementary Table

List of primers used for RT-PCR and ChIP-qPCR

Oligo name	Sequences
Zfp281 NT sgRNA	5' CCCTGTCAATCAAGCGGGCGGTT 3'
Zfp281 CT sgRNA1	5' CCACGAGACCTTACCTGTAACTC 3'
Zfp281 CT sgRNA2	5' CCAATTACATTAGACTCCCAGCC 3'
Brca2 sgRNA	5' CACCGTACACATCTTACAAACCGGG 3'
mouse *Hba2* ChIP-qPCR	Forward：5' CTCCCTTGCACCTGTACCTC 3'
	Reverse：5' CTTTACTGCCCCATGGCTAA 3'
mouse *Nanog* ChIP-qPCR	Forward：5' GGGTAGGGTAGGAGGCTTGA 3'
	Reverse：5' CAGCCTATCTGAAGGCCAAC 3'
mouse *Hoxb13* ChIP-qPCR	Forward：5' GAAGTGTCTGGCGGGTATGT 3'
	Reverse：5' CAAATGCACTGAAAGCCTCA 3'
mouse *Pura* ChIP-qPCR	Forward：5' TGCAGCTAAGGGCTTACCAC 3'
	Reverse：5' CGAAGCAAAAGAGGTGCTG 3'
mouse *Gata6* ChIP-qPCR	Forward：5' TACTGCTCTGCCGGAAAACT 3'
	Reverse：5' GTTTGTTTAGGGCTCGGTGA 3'
mouse *Meis2* ChIP-qPCR	Forward：5' CTTCTTGCCGCTTACTCTCG 3'
	Reverse：5' TTTAGAGCCCCTACGTGGAA 3'
mouse *Kctd1* ChIP-qPCR	Forward：5' TTCCTCTTCCTTCCCACCTT 3'
	Reverse：5' GATTCCGGAGAGTGTGCCTA 3'
mouse *Hoxa1* ChIP-qPCR	Forward：5' AATGCCACTGAAACGGTGAT 3'
	Reverse：5' CGTGACTCTACCAGCCAATG 3'
mouse *Lhx4* ChIP-qPCR	Forward：5' GGAAAATCTCTGCCGTGTGT 3'
	Reverse：5' AGGTCTCTCACCCCAGGTTT 3'
mouse *Vrtn* ChIP-qPCR	Forward：5' TCTGGACGCCTTTTGTCTCT 3'

	Reverse：5' CCTTCCCCCACCTTTTACAT 3'
mouse *Hspd1* ChIP-qPCR	Forward：5' GCACAGAAAACCACATCCCC 3'
	Reverse：5' GGAGTGGATCTGCTCCCAAG 3'
mouse *Pou3f2* ChIP-qPCR	Forward：5' CCCAGCCTAAGAGTGAATGG 3'
	Reverse：5' GGCCTGACTTCAGTCTCCTG 3'
mouse *Actin* RT-qPCR	Forward：5' CCTTCCTTCTTGGGTATGGA 3'
	Reverse：5' ACGGATGTCAACGTCACACT 3'
mouse *Hoxb1* RT-qPCR	Forward：5' GAACCCAGCACTCTCACTCC 3'
	Reverse：5' GTGAAGTTTGTGCGGAGACC 3'
mouse *Hoxb4* RT-qPCR	Forward：5' CACGGTAAACCCCAATTACG 3'
	Reverse：5' TCCTTCTCCAACTCCAGGAC 3'
mouse *Hoxb5* RT-qPCR	Forward：5' GCTTCACATCAGCCACGATA 3'
	Reverse：5' CCAGGGTCTGGTAGCGAGTA 3'
mouse *Gata6* RT-qPCR	Forward：5' CAAAAGCTTGCTCCGGTAAC 3'
	Reverse：5' TGAGGTGGTCGCTTGTGTAG 3'
mouse *Pou3f2* RT-qPCR	Forward：5' TCAAATGCCCTAAGCCCTCG 3'
	Reverse：5' CGGGAGGGGTCATCCTTTTC 3'
mouse *Kctd1* RT-qPCR	Forward：5' ACTAAACGGCTACTGCCACC 3'
	Reverse：5' AGTTCCCGTCGGAGGACATA 3'
