# Supplementary material for: ZFP281-BRCA2 prevents R-loop accumulation during DNA replication
Source: Nat Commun. 2022 Jun 17;13:3493. doi: 10.1038/s41467-022-31211-9 (PMC9205938; doi:10.1038/s41467-022-31211-9)
Supplement: Supplementary file 3 — Reporting Summary [file 41467_2022_31211_MOESM3_ESM.pdf]

nature research

Corresponding author(s): Zhuojuan Luo, Chengqi Lin

Last updated by author(s): May 24, 2022

Reporting Summary

Nature Research wishes to improve the reproducibility of the work that we publish. This form provides structure for consistency and transparency in reporting. For further information on Nature Research policies, see our Editorial Policies and the Editorial Policy Checklist.

Statistics

For all statistical analyses, confirm that the following items are present in the figure legend, table legend, main text, or Methods section.

n/a	Confirmed	
☐	☒	The exact sample size (*n*) for each experimental group/condition, given as a discrete number and unit of measurement
☐	☒	A statement on whether measurements were taken from distinct samples or whether the same sample was measured repeatedly
☐	☒	The statistical test(s) used AND whether they are one- or two-sided<br>*Only common tests should be described solely by name; describe more complex techniques in the Methods section.*
☒	☐	A description of all covariates tested
☐	☒	A description of any assumptions or corrections, such as tests of normality and adjustment for multiple comparisons
☐	☒	A full description of the statistical parameters including central tendency (e.g. means) or other basic estimates (e.g. regression coefficient) AND variation (e.g. standard deviation) or associated estimates of uncertainty (e.g. confidence intervals)
☐	☒	For null hypothesis testing, the test statistic (e.g. F, t, r) with confidence intervals, effect sizes, degrees of freedom and P value noted<br>*Give P values as exact values whenever suitable.*
☒	☐	For Bayesian analysis, information on the choice of priors and Markov chain Monte Carlo settings
☒	☐	For hierarchical and complex designs, identification of the appropriate level for tests and full reporting of outcomes
☒	☐	Estimates of effect sizes (e.g. Cohen's *d*, Pearson's *r*), indicating how they were calculated

Our web collection on statistics for biologists contains articles on many of the points above.

Software and code

Policy information about availability of computer code

Data collection	Images: Zeiss LSM700/ LSM800<br>Western blotting: BioRad Imager<br>Flow cytometry:BD FACS calibur, CellQuest Pro<br>Microsoft Excel 2013<br>Bio-Rad CFX Manager 3.1
Data analysis	Fiji Image J, Zen Light Edition, Microsoft Excel 2013, FlowJo 7.6.1 , Graph Pad Prism 8.0, IGV_2.3.91, Bowtie v2.2.5, MACS v2.1.1, Homer v4.9, deepTools 2.0,R(v3.4.0),<br>1.Clean reads were aligned to the Mus_musculus genome (UCSC genome, mm9) using Bowtie2 (v2.2.5) allowing uniquely mapping reads only.<br>2.Peak calling was performed with MACS2 (v2.1.1).For BRCA2 peaks, associated control samples were used to determine statistical enrichment at p < 1e-5 and FDR < 0.05. For ZFP281 peaks, associated control samples were used to determine statistical enrichment at p < 1e-8 and FDR < 0.05<br>3.Peak annotating was performed with R(v3.4.0) ("ChIPseeker"; "org.Mm.eg.db"; "TxDb.Mmusculus.UCSC.mm9.knownGene"; "clusterProfiler";).<br>4.The motif analysis was performed by Homer (v4.9) with program 'findMotifsGenome.pl' (-len 8,10,12).<br>5. Meta plots were produced by deepTools 2.0 with computeMatrix (reference-point --sortUsing mean --referencePoint center --missingDataAsZero -b 5000 -a 5000).

For manuscripts utilizing custom algorithms or software that are central to the research but not yet described in published literature, software must be made available to editors and reviewers. We strongly encourage code deposition in a community repository (e.g. GitHub). See the Nature Research guidelines for submitting code & software for further information.

Data

Policy information about availability of data

All manuscripts must include a data availability statement. This statement should provide the following information, where applicable:

- Accession codes, unique identifiers, or web links for publicly available datasets
- A list of figures that have associated raw data
- A description of any restrictions on data availability

ChIP-seq data have been deposited in Gene Expression Omnibus (GEO) database under accession number GSE127262 [https://www.ncbi.nlm.nih.gov/geo/query/acc.cgi?acc=GSE127262] and GSE77115 [https://www.ncbi.nlm.nih.gov/geo/query/acc.cgi?acc=GSE77115]. Public ChIP-seq were download from GEO database under accession number GSE24164 [https://www.ncbi.nlm.nih.gov/geo/query/acc.cgi?acc=GSE24164] and GSE12241 [https://www.ncbi.nlm.nih.gov/geo/query/acc.cgi?acc=GSE12241].

Field-specific reporting

Please select the one below that is the best fit for your research. If you are not sure, read the appropriate sections before making your selection.

☒ Life sciences ☐ Behavioural & social sciences ☐ Ecological, evolutionary & environmental sciences

For a reference copy of the document with all sections, see nature.com/documents/nr-reporting-summary-flat.pdf

Life sciences study design

All studies must disclose on these points even when the disclosure is negative.

Sample size	Sample sizes were chosen as large as possible while taking into account the experimental effort required to generate the respective data.
Data exclusions	No data exclusions.
Replication	All experiment were repeated at least three times independently to assure reproducibility. All attempts for replication shows similiar results. Results shown are technical replicates from representative biological replicates.
Randomization	For each experiment, both control and treatments groups were randomly selected.
Blinding	Investigators were blinded to data analysis. Samples were prepared by unblinded. Confocal analysis was performed in blind.

Reporting for specific materials, systems and methods

We require information from authors about some types of materials, experimental systems and methods used in many studies. Here, indicate whether each material, system or method listed is relevant to your study. If you are not sure if a list item applies to your research, read the appropriate section before selecting a response.

Materials & experimental systems

n/a	Involved in the study	
☐	☒	Antibodies
☐	☒	Eukaryotic cell lines
☒	☐	Palaeontology and archaeology
☒	☐	Animals and other organisms
☒	☐	Human research participants
☒	☐	Clinical data
☒	☐	Dual use research of concern

Methods

n/a	Involved in the study	
☐	☒	ChIP-seq
☐	☒	Flow cytometry
☒	☐	MRI-based neuroimaging

Antibodies

Antibodies used

anti-ZFP281, rabbit polyclonal, generated in house
anti-BRCA2, rabbit polyclonal, generated in house
anti-QSER1, rabbit polyclonal, generated in house
anti-H3K4me3, rabbit polyclonal, generated in house
anti-H3K27me3, rabbit polyclonal, Sigma, 07-449
anti-EMSY, rabbit polyclonal, Abcam, ab123
anti-α-tubulin (clone DM1A),mouse monoclonal,Sigma, T9026
anti-PCNA(F-2), mouse monoclonal, Santa Cruz, sc-25280
anti-BrdU (BU-1), mouse monoclonal, Sigma, RPN202
anti-Histone H3 (phospho S10) (mAbcam 14955), mouse monoclonal, Abcam ,ab14955

anti-FLAG (clone M2), mouse monoclonal, Sigma, F3165
anti-V5, rabbit polyclonal, Abcam , ab9116
anti-H3, rabbit polyclonal, Abcam , ab1791
S9.6 (S9.6), mouse monoclonal, Kerafast, ENH001
anti-MEK2, rabbit polyclonal, Proteintech, 11049-1-AP
anti-SUZ12, rabbit polyclonal, Abcam , ab12073
anti-CCNA2, rabbit polyclonal, ABclonal , A2891
anti-CCNC, rabbit polyclonal, ABclonal , A13610
anti-CCNE, rabbit polyclonal, ABclonal , A14225
anti-DDX5 (ABclonal, ARC0575), rabbit monoclonal, ABclonal, A11339
anti-ZFP281, rabbit polyclonal, Abcam, ab112047
ant-γH2A.X(sc-517348), mouse monoclonal, Santa Cruz, sc-517348
anti-phospho-Histone H3 (Thr3) (JY325), rabbit monoclonal, Sigma, 05-746R
Protein A-HRP, Invitrogen, 101023
anti-mouse IgG Alexa fluor 488, Invitrogen, A-11001
anti- rabbit IgG Alexa fluor 546, Invitrogen, A-11035

Validation

All antibodies used have been validated in previous publications/by the manufacturer. References:
anti-ZFP281, rabbit polyclonal, generated in house, Ref: Wang, Y. et al. A permissive chromatin state regulated by ZFP281-AFF3 in controlling the imprinted Meg3 polycistron. Nucleic Acids Res 45, 1177-1185 (2017).
anti-BRCA2, rabbit polyclonal, generated in house, Supplementary Figure 6b, d shows validation of antibodies against QSER1 and BRCA2.
anti-EMSY, rabbit polyclonal, ab123, https://www.abcam.com/EMSY-antibody-ab123.html
anti-QSER1, rabbit polyclonal, generated in house, Supplementary Figure 6a,c shows validation of antibodies against QSER1.
anti-H3K4me3, rabbit polyclonal, generated in house, Ref: Dai, Qian et al. "Striking a balance: regulation of transposable elements by Zfp281 and Mll2 in mouse embryonic stem cells." Nucleic acids research vol. 45,21 (2017)
anti-H3K27me3, rabbit polyclonal, Sigma, 07-449, https://www.sigmaaldrich.cn/CN/zh/product/mm/07449
anti-α-tubulin,mouse monoclonal, Sigma, T9026, https://www.sigmaaldrich.cn/CN/zh/product/sigma/t9026
anti-PCNA, mouse monoclonal, Santa Cruz, sc-25280, https://www.scbt.com/p/pcna-antibody-f-2
anti-BrdU, mouse monoclonal, Sigma, RPN202, https://www.sigmaaldrich.com/US/en/product/sigma/gerpn202
anti-Histone H3 (phospho S10), mouse monoclonal, Abcam ,ab14955, https://www.abcam.com/histone-h3-phospho-s10-antibody-mabcam-14955-ab14955.html
anti-FLAG, mouse monoclonal, Sigma, F3165, https://www.sigmaaldrich.cn/CN/zh/product/sigma/f3165
anti-V5, rabbit polyclonal, Abcam , ab9116, https://www.abcam.com/v5-tag-antibody-ab9116.html
anti-H3, rabbit polyclonal, Abcam , ab1791, https://www.abcam.com/histone-h3-antibody-nuclear-marker-and-chip-grade-ab1791.html?applications=83
S9.6, mouse monoclonal, Kerafast, ENH001, https://www.kerafast.com/productgroup/432/anti-dna-rna-hybrid-s96-antibody
anti-MEK2, rabbit polyclonal, Proteintech, 11049-1-AP, https://www.thermofisher.cn/cn/zh/antibody/product/MEK2-Antibody-Polyclonal/11049-1-AP
anti-SUZ12, rabbit polyclonal, Abcam , ab12073, https://www.abcam.com/suz12-antibody-ab12073.html
anti-CCNA2, rabbit polyclonal, ABclonal , A2891, https://abclonal.com.cn/catalog/A2891
anti-CCNC, rabbit polyclonal, ABclonal , A13610, https://abclonal.com.cn/catalog/A13610
anti-CCNE1, rabbit polyclonal, ABclonal , A14225, https://abclonal.com.cn/catalog/A14225
anti-DDX5, rabbit monoclonal, ABclonal, A11339, https://abclonal.com.cn/catalog/A11339
anti-ZFP281, rabbit polyclonal, Abcam, ab112047, https://www.abcam.com/zfp281znf281-antibody-ab112047.html
ant-γH2A.X, mouse monoclonal, sc-517348, https://www.scbt.com/p/p-histone-h2a-x-antibody-ser-139
anti-phospho-Histone H3 (Thr3), rabbit monoclonal, Sigma,05-746R, https://www.merckmillipore.com/CN/zh/product/Anti-phospho-Histone-H3-Thr3-Antibody-clone-JY325-rabbit-monoclonal,MM_NF-05-746R
Protein A-HRP, Invitrogen, 101023, https://www.thermofisher.cn/order/catalog/product/101023?SID=srch-hj-101023
anti-mouse IgG Alexa fluor 488, Invitrogen, A-11001, https://www.thermofisher.cn/cn/zh/antibody/product/Goat-anti-Mouse-IgG-H-L-Cross-Adsorbed-Secondary-Antibody-Polyclonal/A-11001
anti- rabbit IgG Alexa fluor 546, Invitrogen, A-11035, https://www.thermofisher.cn/cn/zh/antibody/product/Goat-anti-Rabbit-IgG-H-L-Highly-Cross-Adsorbed-Secondary-Antibody-Polyclonal/A-11035

Eukaryotic cell lines

Policy information about cell lines

Cell line source(s)

HEK293T and v6.5 were purchased from ATCC, HEK293 Flp-In TRex (HEK293 FIT) and KH2 were puachased from Thermofisher. WT and EED KO ES cell lines were gifts from Drs. Terry Magnuson and Gang Li.

Authentication

Cells used in this study were validated by RNA-seq and transcriptom comparison with data in public database, such as Encode.

Mycoplasma contamination

Routine mycoplasma tested showed all cell lines were free of mycoplasma contamination.

Commonly misidentified lines
(See ICLAC register)

No commonly misidentified cell lines were used.

ChIP-seq

Data deposition

☒ Confirm that both raw and final processed data have been deposited in a public database such as GEO.

☒ Confirm that you have deposited or provided access to graph files (e.g. BED files) for the called peaks.

Data access links<br>*May remain private before publication.*	GEO database GSE127262 [https://www.ncbi.nlm.nih.gov/geo/query/acc.cgi?acc=GSE127262].
Files in database submission	ChIP-seq of BRCA2 in EED WT mESC;<br>EED WT mESC Input;<br>EED KO mESC Input;<br>ChIP-seq of BRCA2 in EED KO mESC;<br>ChIP-seq of BRCA2 in Non T shRNA mESC;<br>ChIP-seq of BRCA2 in ZFP281 shRNA mESC;<br>ChIP-seq of BRCA2 in ZFP281 WT mESC;<br>ChIP-seq of BRCA2 in ZFP281 KO-1 mESC;<br>ChIP-seq of BRCA2 in ZFP281 KO-2 mESC.
Genome browser session<br>(e.g. UCSC)	Not used

Methodology

Replicates	ChIP-seq experiments were performed in duplicates with an input control for each experiment.
Sequencing depth	ChIP-seq of BRCA2 in EED WT mESC had 49,433,823 reads and 97.02% mapped rate, reads length was 75bp with single-end;<br>EED WT mESC Input had 41,315,880 reads and 97.99% mapped rate, reads length was 75bp with single-end;<br>ChIP-seq of BRCA2 in EED KO mESC had 40,943,175 reads and 91.38% mapped rate, reads length was 75bp with single-end;<br>EED KO mESC Input had 71,546,466 reads and 93.17% mapped rate, reads length was 75bp with single-end;<br>ChIP-seq of BRCA2 in Non T shRNA mESC had 7,053,829 reads and mapped 96.67% rate, reads length was 150bp with paired-end;<br>ChIP-seq of BRCA2 in ZFP281 shRNA mESC had 10,739,096 reads and 96.84% mapped rate, reads length was 150bp with paired-end;<br>ChIP-seq of BRCA2 in ZFP281 WT mESC had 12,616,329 reads and 55.34% mapped rate, reads length was 150bp with paired-end;<br>ChIP-seq of BRCA2 in ZFP281 KO-1 mESC had 11,187,999 reads and 53.32% mapped rate, reads length was 150bp with paired-end;<br>ChIP-seq of BRCA2 in ZFP281 KO-2 mESC had 9,494,452 reads and 53.92% mapped rate, reads length was 150bp with paired-end.
Antibodies	anti-BRCA2, homemade
Peak calling parameters	macs2 callpeak -control.bam -t treatment.bam -m 5 50 -p 1e-5 -f BAM -B -g mm -n BRCA2_out
Data quality	Signal tracks for each sample were generated using the deepTools'BamCoverage command (v3.5.1) and were normalized to Counts Per Million mapped reads (CPM) for visualization.
Software	Clean reads were aligned to the Mus_musculus genome (UCSC genome, mm9) using Bowtie2 (v2.2.5) allowing uniquely mapping reads only.Peak calling was performed with MACS2 (v2.1.1).

Flow Cytometry

Plots

Confirm that:

☒ The axis labels state the marker and fluorochrome used (e.g. CD4-FITC).

☒ The axis scales are clearly visible. Include numbers along axes only for bottom left plot of group (a 'group' is an analysis of identical markers).

☒ All plots are contour plots with outliers or pseudocolor plots.

☒ A numerical value for number of cells or percentage (with statistics) is provided.

Methodology

Sample preparation	Cells were pulsed with 10 μM EdU for 30 min. Cell cycle analysis were performed using the BeyoClick™ EdU Cell Proliferation Kit with Alexa Fluor 488 (Beyotime, C0071S) according to the manufacture's instruction.Then cells were harvested, washed with PBST (0.5% Tween-20), incubated with RNase A for 30 min at 37 °C, and stained with 50 μg/mL propidium iodide (PI) for 30 min, then cell suspensions were filtered through 40um cell strainer.
Instrument	BD FACS Calibur
Software	CellQuest Pro software was used for data collection and FlowJo 7.6.1 was used for data analysis.

Cell population abundance	For the cell cycle, there are ~20.3% of G1/G0 phase cell, ~67.9% of S phase cell and ~11.8% of G2/M phase cell in WT group, ~24.8% of G1/G0 phase cell, ~61.2% of S phase cell and ~14.0% of G2/M phase cell in ZFP281 KO 1 group,~26.3% of G1/G0 phase cell, ~60.7% of S phase cell and ~13.0% of G2/M phase cell in ZFP281 KO 2 group,
Gating strategy	Single mESC cells were gated with EdU and PI. EdU and PI low cells were resident G1/G0 phase cells; EdU high and PI middle cells were resident S phase cell cells; EdU low and PI high cells were resident M phase cell cells.

☒ Tick this box to confirm that a figure exemplifying the gating strategy is provided in the Supplementary Information.
